# Official social media and its impact on public behavior during the first wave of COVID-19 in China

**DOI:** 10.1186/s12889-022-12803-y

**Published:** 2022-03-03

**Authors:** Huan Liu

**Affiliations:** School of Journalism and New Media, Xi’an Jiaotong University, Xi’an, China

**Keywords:** Information quality, Risk perception, Behavior intention, Continued use, COVID-19

## Abstract

**Background:**

During the COVID-19 pandemic, official social media became a critical channel for the public to obtain pandemic information. No matter the positive function or negative effect of information dissemination, it involves the public’s risk perception and behavior. This study was designed to contribute to the existing research on how official social media information quality (IQ) and risk perception (RP) affect preventive behavior (PB) and continued use behavior (CB) of official social media during the first wave of COVID-19.

**Methods:**

The required data were extracted from a national online survey of the Chinese Mainland during March 24–30 2020, a random sample was asked to participate in the survey (n = 666). Data analysis was performed using regression analysis, structural equation modeling, mediating effect analysis, and one-way ANOVA analysis.

**Results:**

The results show that IQ (10.010 ± 3.568) has direct and indirect positive impact on PB (9.475 ± 3.571), and has a low significant positive indirect impact on CB (3.739 ± 1.566). The IQ has a significant positive impact on RP (β = 0.548), which show that there is no “risk perception paradox” in COVID-19. Furth more, this study also provides new evidence indicating that RP mediates the relationship between IQ and PB. According to the region, gender, age and annual income, and there are significant differences in PB and CB.

**Conclusion:**

The study findings have remarkable implications for improving the information quality and public behaviors. Too high or too low level of risk perception is not conducive to pandemic prevention and control. Official social media should indirectly affect information flow through the reasonable supply of pandemic information and constantly improve the quality of pandemic information to avoid public’s undue panic and excessive health concerns during this ongoing outbreak and subsequent national public emergency events.

## Introduction

The World Health Organization declared the spread of COVID-19 pandemic a global public health emergency. The WHO’s 13^th^ Coronavirus Report, released that excess of true and false information both online and offline, which makes it difficult for citizens to find trusted sources and reliable guidance when needed. During the COVID-19 pandemic, the infodemic, even though some misinformation may just be confusing, many false and misleading claims such as those about fake or questionable cures, or incorrect recommendations about prevention or public behavior can be harmful to life and can exacerbate the outbreak [1]. Understanding governmental pandemic prevention measures is a critical predictor of public risk perception [2], particularly should protect the elderly [3]. Public risk perception plays an important role in the response to health emergencies, public health policies, affecting risk management and risk communication strategies [4], and in the adoption of these actions, people’s feelings, and their daily habits [5]. However, the change in daily habits, the limitation of social life and the risk of COVID-19 could have an impact on the well-being of individuals [6].

Individuals communicate with others have cyber-based and place-based information sources, who with higher levels of perceived cyber-based information overload have greater stress, poorer health, and less time devoted to contemplative activities [7]. During the outbreak of infection diseases, particularly when traditional media do not provide relevant, timely information for the public, less credible information from Public Health Officials [8], and tele education of family health ambassadors [9] and individuals use social media as an effective tool and immediate information source for communicating relevant information with others [10]. As COVID-19 spreads rapidly, public fear of the unknown risks of the pandemic and the relative authority of official social media became an essential public messenger and mode to obtain and disseminate pandemic information.

Accordingly, the present research was designed to contribute to the prior research with the media use motivations [11–13], risk perception [14, 15], and the intention to adopt information [16]. Studies found that personal experience and trust in expert authorities have the greatest impact on risk perception between personal experience and ready to take protective actions, a risk perception paradox exists in that it is assumed that high risk perception will lead to personal preparedness and, in the next step, to risk mitigation behavior [17], and can result in heavy losses to individuals and society. This study was designed to contribute to the existing research on how official social media information quality (IQ) and risk perception (RP) affect preventive behavior (PB) and continued use behavior (CB) of official social media during the COVID-19 outbreak. Specially, this study investigates three issues: (1) Is there “risk perception paradox” in the context of COVID-19 pandemic? (2) How QI affects PB and CB? (3) Is there difference in public behavior in different regions, genders, ages and annual household income? The answer to these questions will provide empirical evidence for future risk governance and communication, improving public intention to act against the pandemic, and the information management ability of official social media operation institutions.

## Literature review and research hypotheses

The protective action decision-making fuses to promote risk communication activities in a better way. In this process, the degree of risk exposure, the quality of risk information, the perception of protection activities, and stakeholders’ perceptions, which affect public decision-making on protective actions [18, 19]. Social media is “a double-edged sword”, if used appropriately, can increase the public risk perception through two self-related emotions (fear and anger), and significantly enhance their preventive behaviors [20]. In addition, if used excessively, or misinformation disseminated on the social media, risk perception can be unnecessarily exaggerated through the lens of social media [21] [22].

According to the Social Amplification of Risk Framework, the social media can function as a “social amplification station’ to form the public risk perception [23]. The “vertical” official information source and formal social interaction can influence public risk perception [24]. There is significant uncertainty in the public risk perception of infectious disease outbreaks [25]. Appropriate preventative or individual avoidance behaviors rely on risk perceptions [26]. The structure of the health belief model predicted public’s perceived stress and risk during COVID-19 [27, 28].

### Official social media information quality and public behavior

The expression and reception of infectious disease risk information on social networking sites can affect public preventative behaviors [10]. The input of limited data and the illusion of risk control lead to the cognitive bias of individuals, which leads to the difference in the degree of risk cognition [29]. Protection awareness, stakeholders, and risk awareness can positively affect individuals to take protective measures, and risk perception plays a partially mediating role [30]. Faced with the rapid spread of COVID-19 and a crisis of prevention and control, official social media at all levels released different types of pandemic information. Some channels released professional or scientific information, associated dangers of COVID-19 to reduce the panic. Some channels published specific protective measures and touching stories of local government at all levels to let the public know and understand the herculean efforts made by governments and medical workers. Some channels released the itinerary of confirmed cases and asked close contacts of those confirmed to be under quarantine and medically observed to ensure their health and safety and prevent the spread of COVID-19. In this way, the quality of information released by official social media will affect public intention to take preventive actions and continued use of official social media. Based on these factors, I proposed the following hypotheses:

- **H1a**: The IQ has a significant impact on PB.
- **H1b**: The IQ has a significant impact on CB.

### Official social media information quality and risk perception

News media exposure correlates positively with the cognitive dimensions of risk characteristics [31]. The news information that causes fear correlates positively with the risk perception of individuals. Fear-inducing news information leads to people talking about risk directly or indirectly through perceived risk. Risk perception appears to be more closely related to the intention to talk about risk at the individual level than at the social level [32]. Risk perception can be influenced by the sensationalistic headline, emotional processing (evaluation of the consequences), and information quality than a logical argument [33, 34]. The systematic analysis and release of risk information brought by COVID-19 through official social media can serve as a public risk alert and education. The official social media information quality on how to prevent the pandemic affects public risk perception of the pandemic. I proposed the following hypothesis based on this concept:

- **H2**: The IQ has a significant impact on RP.

### Risk perception and behavior intention

When the available risk information is not enough to make them take preventive behavior, people will search for health information to solve the uncertainty of risk [18]. Perceived risk to disaster also led the public to seek and process information to alleviate anxiety, and risk perception towards emergencies can influence their subsequent mitigation intentions and actions [35]. Risk perception is the most direct public psychological response to the pandemic, and it shows regional differences and demographic characteristics, and risk perception is affected by information quality. Furthermore, it affects social and economic psychology, mental health, and public behavioral intention. Risk perception has a significant positive impact on Europeans’ flood control behaviors, and women have a higher level of risk perception [36]. Risk perceptions were important drivers for the acceptance of the government’s implemented measures to control COVID-19 and for more preventive behavior (i.e., keep social distance and more hygienic behavior) [37].

Although risk perception plays a mediating or social amplifying role between personal experience and intention to take preventive actions, there is still a risk perception paradox phenomenon that individuals with high-risk perception still do not take preventive action. In the context of COVID-19, most of the public strictly abides by the pandemic prevention and control regulations. Still, some people remain who perceive a high degree of risk and have the mindset that “they cannot be infected.” Fortunately, they are a minority group. In other words, the higher level of risk perception, the stronger the public intention to take positive preventative action. The higher the public's risk perception, the more frequently they use official media to obtain authoritative pandemic information. Based on this assumption, I proposed the following hypotheses:

- **H3a**: RP has a significant impact on PB.
- **H3b**: RP has a significant impact on CB.

Spatial distance will affect public sensitivity to risk perception, increased proximity increases risk perception [38]. When faced with risk events, women have a higher level of risk perception than men, and the age affects individual risk experience and behavior intention [39]. The potential harm of misinformation could be more substantial for low-income countries than high-income countries [40], where low health literacy levels, poor health infrastructure and poor resource settings exist [41].

- **H4a**: There is significant difference in PB with different risk levels.
- **H4b**: There is significant difference in CB with different risk levels.
- **H5a**: There is significant difference in PB between males and females.
- **H5b**: There is significant difference in CB between males and females.
- **H6a**: There is significant difference in PB at different ages.
- **H6b**: There is significant difference in CB at different ages.
- **H7a:** There is significant difference in PB with different annual household incomes.
- **H7b:** There is significant difference in CB with different annual household incomes.

## Methods

### Research Model

**Fig. 1:**
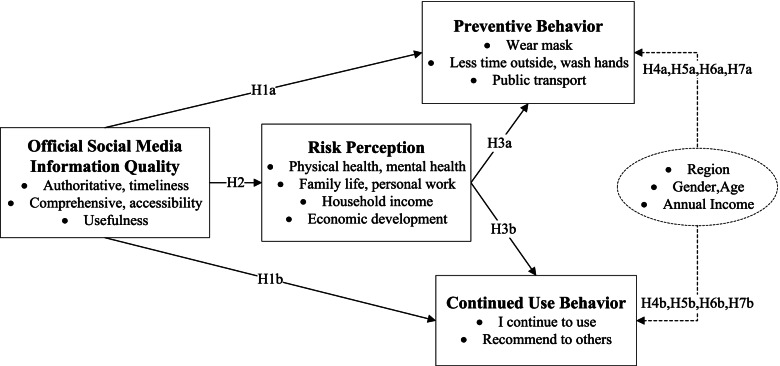
Conceptual Model and Summary of the research hypotheses

Considering the abovementioned arguments on the relationships among IQ, RP, and preventive and continued use behaviors, the conceptual model applied in this paper for the examination of research hypotheses has been showed in Fig. 1.

### Instruments and measures

The sample was the survey data of the Chinese Mainland during the period of March 24–30 2020. IQ, RP, PB and CB were also measured using the 5-point Likert scale. Taking China’s specific situation into account, and I translated all the items into Chinese. From this online panel, a random sample was asked to participate in the survey, primarily through the combination of Wechat, QQ, and Internet to fill questionnaire (in Chinese) anonymously. Since this study focused on the impact of official social media information quality on public behaviors, the questionnaire set the item “whether you use official social media to obtain information of the COVID-19 or not”, and finally selected respondents who answered “yes” as the analysis sample. Finally, a total of 666 valid samples were obtained. The demographic profile of the survey respondents is presented in Table 8.

#### Official social media information quality (Mean = 10.090, SD = 3.568, Cronbach’s = 0.832)

Measurement of IQ was carried out employing a 5-point Likert scale (1 = strongly disagree, 5 = strongly agree) through asking the respondents’ perception of COVID-19 information via official social media during the COVID-19 outbreak: (1) I think the information released by official social media is authoritative; (2) I think the information released by official social media is timeless; (3) I think the information released by official social media is comprehensive; (4) I think the information released by official social media is accessibility; (5) I think the information released by official social media is usefulness. I arranged the five items to create an index for IQ. These items were adapted and modified based on the literature [42–44].

#### Risk perception (Mean = 15.222, SD = 5.090, Cronbach’s = 0.859)

Measurement of RP in this study was carried out employing a 5-point Likert scale (1 = strongly disagree, 5 = strongly agree) through asking how much the respondents agreed with the following seven risk statements directly to COVID-19: (1) I have felt that COVID-19 is dangerous to my physical health; (2) I have felt that COVID-19 is dangerous to my mental health; (3) I am worried that COVID-19 would affect my job; (4) I am worried that COVID-19 would affect my household income; (5) I am worried that COVID-19 would affect my family life; (6) I am worried that COVID-19 would affect children’s growth; (7) I am worried that COVID-19 would affect economic development. I arranged the seven items to create an index for RP. Similarly, these seven items were adapted and modified based on the literature [31, 45].

#### Preventive behaviors (Mean = 9.475, SD = 3.571, Cronbach’s = 0.819)

Measurement of respondents’ intention to be engaged in preventive and continued use activities was carried out of a 5-point Likert scale (1 = strongly disagree, 5 = strongly agree), according to which the respondents were asked how much they engaged in the following preventive behaviors such as masking-wear, physical distancing, etc., since the first COVID-19 patient was confirmed: (1) I have worn a mask to reduce the risk of COVID-19 infection; (2) I have reduced outdoor activities, such as going department stores, walking, etc.; (3) I have tried not to take public transportation; (4) I have tried to wash my hands or used hand sanitizer more often to prevent the risk of COVID-19 infection; (5) I have tried to leave the worst-infected regions to the other lower-infected regions. I arranged the five items to create an index for PB. These five items were adapted and modified based on the literature [39, 46].

#### Continued use behaviors (Mean = 3.739, SD = 1.566, Cronbach’s = 0.673)

Measurement of CB was also carried out employing a 5-point Likert scale (1 = strongly disagree, 5 = strongly agree), according to which the respondents were asked how much they engaged in the following continued use behaviors: (1) I continue to use official social media; (2) I recommend others to use official social media. I arranged the two items to create an index for CB. These items were adapted and modified based on the literature [47].

#### Dataset distribution

**Fig.2:**
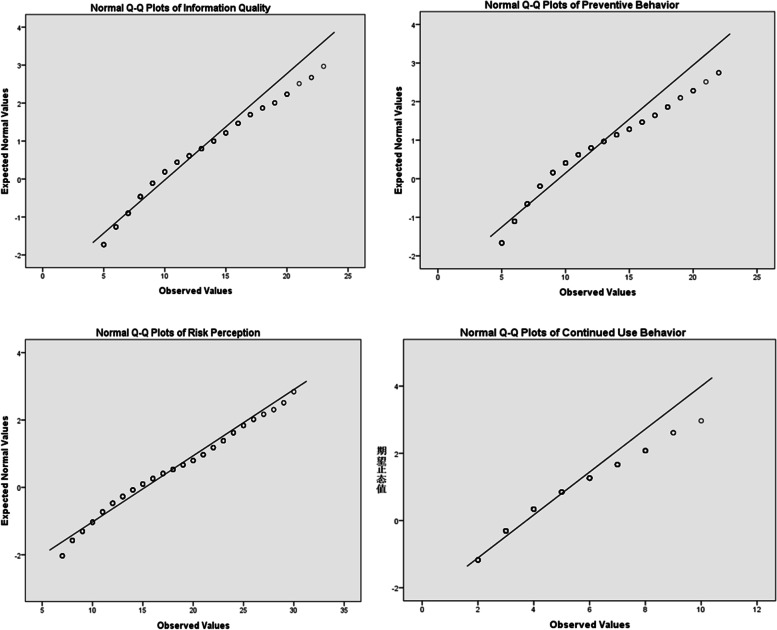
Normal Q-Q Plots of all variables

Previous studies proposed an alternative univariate normality test to the Jarque–Bera test [48]. The proposed statistic is based on the sample second power Skewness and Kurtosis, while the Jarque–Bera statistic uses sample Pearson’s Skewness and Kurtosis that are the third and fourth standardized sample moments, respectively [49]. When testing whether dataset has normal distribution characteristics or not, Shapro-Wilk test is recommended for small samples (less than 50), and Kolmogorov–Smirnov test is recommended for large samples (more than 50), so this study indicated the Kolmogorov–Smirnov test (in Table 1). The requirement for a perfectly normal distribution is very difficult to meet. If the absolute vale of Kurtosis is less than 10 and absolute value of Skewness is less than 3 [50], indicating that the dataset can be accepted as approximately normally distributed. The results showed that absolute values of Kurtosis of all variables were less than 10, and absolute values of Skewness of all variables were less than 3 (in Table 1), and the Normal Q-Q Plots of all variables (in Fig. 2) showed that observed values and expected normal values of all variables were almost in a straight line, so the dataset of this study could be accepted as approximately normally distributed.

**Table 1:** Normality Test

Variable	Mean	Standard	Skewness	Kurtosis	Kolmogorov–Smirnov	
					W	P
Information Quality	10.090	3.568	0.811	0.304	0.938	0.000
Risk Perception	15.222	5.090	0.460	-0.542	0.964	0.000
Preventive Behavior	9.475	3.571	1.085	0.754	0.901	0.000
Continued Use Behavior	3.739	1.566	1.002	0.708	0.881	0.000

## Results

### Reliability and validity tests

#### Reliability test

Cronbach’s alpha is a popular method to measure the reliability of a score to summarize the information of several items in questionnaires, but the alpha coefficient might be non-robust [51]. This study proposed Class Correlation Coefficient and 95% confidence interval from samples to estimate alpha [52]. SPSS24.0 statistical analysis software was used to test the internal consistency of the items. The results showed that the Cronbach’s alpha of the total table = 0.918 > 0.70 and the Cronbach’s alpha coefficients of each subscale (IQ, RP, PB, and CB were 0.832, 0.859, 0.819, and 0.673) (in Table2). All Cronbach’s alpha coefficients were higher than the reference value of 0.60, and the 95% confidence interval of all variables didn’t include 0, indicating that the quality of questionnaire survey data was reliable.

**Table 2:** Reliability Test

Scale	Cronbach’s alpha	Class C	LLCI	ULCI
Total Scale	0.918	0.382	0.355	0.412
*Subscales*	*—*	*—*	*—*	*—*
Information Quality (IQ)	0.832	0.498	0.463	0.535
Risk Perception (RP)	0.859	0.466	0.433	0.500
Preventive Behavior (PB)	0.819	0.531	0.493	0.568
Continued Use Behavior (CB)	0.673	0.507	0.448	0.561

Class C., Class correlation coefficient, is the correlation coefficients defined by consistency are used. The variance between measures is excluded from the denominator variance

### Validity test

#### Confirmatory factor analysis

**Fig. 3:**
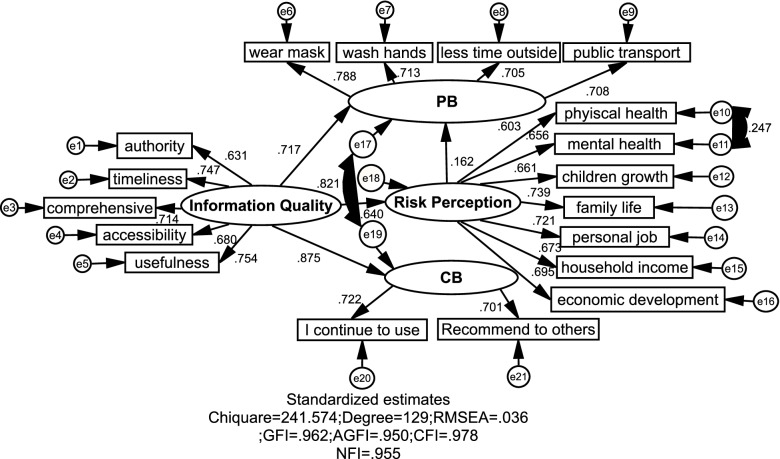
Path analysis result

Application of AMOS software version 22.0 aimed at Confirmatory Factor Analysis (CFA). The standard load coefficient of each measurement item was > 0.60, and the corresponding coefficient was significant at the level of 0.05 (in Table 3). The value factor loading shows the correlation between factors (latent variables) and measurement items (explicit variables) [53]. In terms of the measurement relationship: For Preventive Behavior, the absolute value of the standardized loading coefficient is 0.419 < 0.6 in the measurement of Leave the worst-affected areas, which indicated that the measurement relationship is weak. It can be considered to remove the measurement relationship (showed in Fig. 3) and make other analysis.

**Table 3:** Confirmatory Factor Analysis

Variable	Items	NFL	S.E	z	p	SFL
Information Quality	Authority	1.000	—			0.631
	Timeless	1.039	0.084	15.636	0.000	0.748
	Comprehensive	1.266	0.084	15.119	0.000	0.715
	Accessibility	1.220	0.084	14.557	0.000	0.680
	Usefulness	1.299	0.083	15.722	0.000	0.754
Risk Perception	Physical health	1.000	—			0.636
	Mental health	1.069	0.073	14.657	0.000	0.684
	Children growth	1.065	0.074	14.360	0.000	0.666
	Family Life	1.184	0.076	15.532	0.000	0.738
	Personal Job	1.119	0.074	15.114	0.000	0.711
	Household Income	1.031	0.072	14.323	0.000	0.664
	Economic development	1.046	0.071	14.657	0.000	0.684
Preventive Behavior	Leave the worst-affected	1.000	—			0.419
	Wear mask	1.792	0.170	10.438	0.000	0.781
	Wash hands	1.545	0.150	10.139	0.000	0.712
	Less time outside	1.536	0.154	10.076	0.000	0.699
	Public Transport	1.469	0.148	10.031	0.000	0.711
Continued Use Behavior	I continue to use	1.000	—			0.719
	Recommend to others	0.970	0.058	16.756	0.000	0.705

*NLF* Non-standardized Factor Loading, *SFL* Standardized Factor Loading

The value of composite reliability (CR) was calculated for examination of the instrument reliability and its internal consistency. All the CR values were > 0.7, which has good internal consistency. Pearson product-moment correlation is used to analysis a linear relationship between 2 continuous variables, and is typically used for jointly normally distributed [54]. The variables of IQ, RP, PB, and CB were all measured by 5-Likert scales, and they were approximately normally distributed (in Table1 and Fig. 2), and they were analyzed by Pearson product-moment correlation. The square root values of Average Variance Extracted (AVE) of each variable are greater than the Pearson product-moment correlation coefficients between this and other variables (in Table 4). It indicates that the questionnaire has good discriminant validity.

**Table 4:** Pearson correlation and AVE square root value

Variable	CR	1	2	3	4
1. Information Quality	0.882	0.774			
2. Risk Perception	0.893	0.541***	0.737		
3. Preventive Behavior	0.858	0.692***	0.530***	0.745	
4. Continued Use Behavior	0.859	0.651***	0.443***	0.701***	0.868

N = 666; the diagonal line is the square root value of AVE, and the other values are the correlation coefficients between variables; **p < 0.05, ***p < 0.01

### Path analysis and hypothesis testing results

The chi-square value and the degree of freedom ratio (1 < chi-square/df < 2) were used to judge the fitness of the hypothetical model and the sample data in this study. The results are presented in Table 5 and Fig. 3. All the fit indicators satisfied the acceptable range recommended in the previous studies [55]. For the default model, the normed chi-square (CMIN/DF) = 1.873 < 2 indicated that the fitness of hypothetical model and sample data was accepted.

**Table 5:** CMIN

Model	NPAR	CMIN	DF	p	CMIN/DF
Default model	42	241.574	129	0.000	1.873
Saturated model	171	0.000	0		
Independence model	18	5332.347	153	0.000	34.852

CMIN chi-square of model

The results show that the path coefficient between risk perception (RP) and continued use behavior (CB) was not significant, so I deleted this path. Combined with the revised indicators in the output results, it was necessary to establish the covariation relationship between the error terms of preventive behavior (PB) and continued use behavior (CB), and between the error terms of physical health and mental health, and estimate the revised model. The model estimation results (Fig. 3) showed that the fitting indexes of the modified model were: χ^2^/df = 241.574/129 = 1.873 < 2, GFI = 0.962 > 0.9, AGFI = 0.950 > 0.9, RMSEA = 0.036 < 0.05, CFI = 0.978 > 0.9 and NFI = 0.955 > 0.9. All the indexes satisfied the acceptable range. The above results showed that the covariance matrix of the modified hypothesis model fit well with the sample data.

The analysis results for the four main constructs are showed in Table 6 and Fig. 3. IQ has high significant direct and indirect impacts on PB (β = 0.804 & β = 0.717), and IQ has a high significant direct impact on CB (β = 0.791), which support the H1a and H1b. IQ has a direct impact on RP (β = 0.548), which support the H2. RP has a high significant direct impact on PB (β = 0.212), which support the H3a. The Hypothesis H3b is not supposed, because the IQ has a low significant indirect on CB (in Table 7). Eventually, path analysis was used to examine the overall impacts of IQ on RP and preventive behaviors and continued use behaviors.

**Table 6:** Weighted Regression Coefficients

	Estimate	S.E	C.R	p-value
H2: Information Quality → Risk Perception	0.548***	0.048	11.522	0.000
H1a: Information Quality → PB	0.804***	0.062	12.995	0.000
H1b: Information Quality → CB	0.791***	0.051	15.570	0.000
H3a: Risk Perception → PB	0.212***	0.057	3.724	0.000

^*^*p < 0.05, ***p < 0.01. Estimation, non-standardized coefficients; *CR* critical ratio

**Table 7:** Mediating Effect Result

Mediating path	LLCI	ULCI	Mediating effects
Information Quality → Risk Perception → PB	0.085	0.166	0.120***
Information Quality → Risk Perception → CB	0.015	0.047	0.031***

^*^*p < 0.05, ***p < 0.01

### Mediating effect

According to what was mentioned and the analysis results (in Table 7) of the mediating effect. The Sobel tests were carried out by using the Process procedure in SPSS24.0. The results showed that RP play a partial mediating role in the relationship between the IQ and PB, and the mediating effect is 0.120. The results also showed that RP have a low significant impact on the relationship between the IQ and CB, when the mediating effect is 0.031, which can be neglected.

### One-way Analysis of Variance (ANOVA)

To understand the difference between PB and CB in the context of COVID-19, a one-way ANOVA was conducted, and the results were showed (in Table 8). ANOVA uses the statistic F, which is the ratio of between and within group variances. ANOVA is focused on the differences of group means, and the differences of variances [56]. ANOVA methods required continuous and normal data, homogeneous variances, and independence between groups, etc.[57]. The group variables (region, gender, age, income) were measured by one-choice question, which ensured the independence between different groups. The dependent variables (PB and CB) were measured by the 5-Likert scale, and were continuous data and approximately normally distributed (in Table 1 and Fig. 2). Meanwhile, this paper carried out the homogeneity test of variance, and the results showed that the variances of all independent groups had no significant difference. The dataset satisfied the requirements of numerous assumptions of ANOVA, and they were analyzed by one-way ANOVA.

**Table 8:** Difference in Public Behavior

Variable	PB				CB		
	Sample	Mean	F	p	Mean	F	p
**Region**							
High-risk	141	10.206	7.752***	0.006	4.184	14.789***	0.000
Low-risk	525	9.2781			3.619		
**Gender**							
Female	284	9.919	7.749***	0.006	3.912	6.107**	0.014
Male	382	9.144			3.610		
**Age**							
Under 18 years old	29	8.483	9.910***	0.000	3.517	4.810***	0.001
18–35 years old	413	9.022			3.564		
36–45 years old	180	10.283			4.050		
46–60 years old	39	10.436			4.154		
60 + years old	5	9.476			3.739		
**Annual Income**							
Less than 30,000	293	8.440	36.433***	0.000	3.277	28.697***	0.000
30,000–100,000	269	9.487			3.822		
100,001–200,000	85	12.588			4.835		
More than 200,000	19	11.316			4.780		

^*^*p < 0.05, ***p < 0.01

There were significant differences in the PB and CB between high-risk and low-risk regions. The PB and CB in high-risk regions were higher than those in low-risk regions. There was a significant difference in the PB and the CB between men and women. Women’s PB and CB were higher than men’s. There were notable differences in the PB and CB at different ages, and the PB of those aged 46–60 was the strongest, and the CB of age 36–45 was the strongest. There were notable differences in the PB and CB at different income levels. All the results abovementioned support the H4a, H4b, H5a, H5b, H6a, H6b, H7a and H7b.

## Discussion

This paper contributes to our knowledge of how the official social media information affects preventive and continued use behavior during the global pandemic of the COVID-19. Recent research has mainly devoted efforts to explain the risk perception of pandemic [2, 14, 26, 30, 31, 58], whereas relative few studies have explored how information of official social media influenced public’s behavior through the risk perception during COVID-19. However, information quality online, which can impact the citizens’ recognition and risk perception of pandemic around the world, and provide insight into how to respond to the COVID-19 pandemic and the other future pandemics [59, 60], but official social media has received little attention.

The IQ has a high significant direct and indirect impacts on the PB through RP, and the IQ has a direct impact on CB. RP has a high significant direct impact on public behavior, which has been approved in previous studies on COVID-19 [61]. Currently, due to the rapid growth of social media, coupled with the repeated COVID-19 outbreak, the duration is lengthier, reducing the level of public risk perception of the pandemic, leading to paralysis by the public on pandemic preventive ideas [17]. It indicated that media was the one of many factors that affected public’s risk perception, but the judgment of individual risk perception depended on the information quality from personal experience. The authority, accessibility, comprehensive, timeless, usefulness of information quality, as well as the characteristics of information source and channels (e.g., official social media) positively affected the public’s risk perception. The COVID-19 had the characteristics of serious threat and wide scope of influence, and official social media reported generally respond objectively to the pandemic situation and progress in pandemic prevention and control, and the risk of COVID-19 was objective. When the magnitude of pandemic information is overwhelming in receiving and processing, automatic filtering may occur, which will harm public health and pandemic prevention. From this perspective, the higher official media information quality of COVID-19 is expected to increase the public RP.

This paper found that in the first wave of COVID-19, the public’s risk perception was not only significant influenced by the official social media information quality, but also had a high significant positive impact on the preventive behavior, that is, the risk perception had a significant mediating impact on the relationship between official social media and preventive behavior. The implication is that information managers in official social media and public health emergencies need to observe changes in people’s risk perception in real time. Too high or too low risk perception is not conducive to pandemic prevention and control, and indirectly affects information flow through reasonable supply of media information.

In the case of widespread public health emergencies such as the COVID-19, about which individuals have no enough and authoritative information, they tend to trust in official social media and governments [62]. Lower quality messages would not meet individuals’ needs in the risk and make people turn elsewhere [63]. The determinants of risk perception and behaviors are media information features and their processing by the receivers [64]. This means that official social media should need to constantly improve the quality of pandemic information.

First, the operation departments of official social media should analyze the time characteristics and critical dimensions of public attention to the COVID-19 pandemic based on the science and evidence. Combining with the priority and urgency of pandemic information, and must reach public and enable them to make informed decisions on how to protect themselves and their communities in a health emergency [65], to avoid the public miss essential data due to emotional fatigue and information overload. Additionally, public feedback mechanisms should be implemented to support interactive communication and promptly clarify rumors related to the COVID-19 pandemic. Second, Coordinated work and partnering with a variety of stakeholders, is required to ensure the availability of information via informal social media [1]. Third, the local governments and operations department of official social media should reach out to key communities to ensure their concerns and information needs are understood. Meanwhile, information sources and strategic partnerships should be established across all sectors, including but not limited to official social media, and public health authorities, academia, technology sectors, the food and agricultural sector, health care, hospital and medical professional associations. On the other hand, risk perception can be seen as an emotional response, and individuals show higher likelihood to carry out behaviors in the situation of stronger emotions, with higher quality of the warning information [66]. The perceived information quality, information sufficiency and emotions (perceived threat) are determinants of the behaviors [67]. As it is found, judging the public information preference in time and the changing trend of risk perception and behavior, and adjust the release of information based on timeliness, would improve public intention to act against COVID-19 and continued use of official social media.

This study showed that the public behavior was also significant influenced by demographic characteristics, such as gender, age, and annual income. According to pandemic risk degree, user’s features, including age, gender differences and income differences, the public behavior were also different. For example, in many risk regions, risk communication activities were mainly targeted at men, who may experience less fear than women, and may decide to take fewer or no preventive measures. It is recommended that risk communication involve more women as this may increase the likelihood of preventive actions. Official social media should publish different and precise pandemic information, and warrant a pre-emptive strategy for busting misinformation and indicate a higher demand for localized fact checks in these countries and a public belief, especially in low-income countries [40]. It will improve users’ interest and focus on pandemic information, and avoid undue panic and excessive health concerns. Improving digital literacy and increasing fact-checking capacities, supporting and facilitating people to think more critically about the relationship between information and their health is one potentially powerful way of intervening in and reshaping cultural norms around how I consume information and how I understand its impacts on our lives [68].

This study has some limitations. In the context of COVID-19, there are complex influencing factors on public behavior. Although I explored the influence relationship and formation mechanism of official social media information quality on public behavior, I did not consider the interactions between official social media and the public. Future research could use mining tools to conduct in-depth analysis and further probe the influence mechanism of public comments on pandemic information content, the public and governmental interactive content on public behavior, to improve pandemic prevention behavior and continued use of official social media.

## Conclusion

It is crucially important for official social media and the other authorities, and the public to have access to the right information, at the right time and right platforms. Responses to the COVID-19 pandemic and related misinformation require systematic and coordinated action from stakeholders of government and society, and official social media promote authoritative information and fight misinformation, for inaccurate information may cause public panic responded in a timely manner, to guide the public correctly handling the speculation and rumors in the COVID-19 outbreak. This requires timely translation of evidence into knowledge that public can acquire, adapted to their needs and characteristics. I call on public to demand evidence-based and official information, and take actions to improve digital literacy and increase fact-checking capacities, and use trusted information to protect themselves and the most vulnerable. Government health agencies and official social operators should regularly update information of the pandemic in a focused manner to minimize uncertainty and ensure that public sentiment can be calmed and preventive behavior. can be improved in a timely manner.

## Data Availability

The data presented in this study are available on request from the corresponding author. The data are not publicly available due to regulations and guideline of data open policy according to the National Social Science Foundation of China.
